# Graphene-, Transition Metal Dichalcogenide-, and MXenes Material-Based Flexible Optoelectronic Devices

**DOI:** 10.3390/nano16010025

**Published:** 2025-12-24

**Authors:** Yingying Wang, Geyi Zhou, Zhisheng Zhang, Zhihong Zhu

**Affiliations:** School of Advanced Interdisciplinary Studies, National University of Defense Technology, Changsha 410073, China; wanghappyingying@163.com (Y.W.); 19989687707@163.com (G.Z.); 17708512413@163.com (Z.Z.)

**Keywords:** two-dimensional (2D) materials, flexible, optoelectronic devices

## Abstract

Characterized by their atomic thickness and exceptional mechanical properties, two-dimensional (2D) materials offer a compelling platform for developing flexible optoelectronic devices that maintain performance stability under mechanical deformation such as bending and stretching. This review systematically summarizes and critically discusses the recent advancements in applying three prominent 2D material categories—graphene, transition metal dichalcogenides (TMDs, e.g., MoS_2_ and WS_2_), and MXenes—in flexible optoelectronics. We focus on their specific applications in flexible photodetectors, light-emitting devices, optical modulators, solar cells, and gas sensors. A particular emphasis is placed on analyzing the unique physicochemical properties of these materials and elucidating the underlying mechanisms that enable bandgap stability and efficient optoelectronic conversion under mechanical strain. The potential of these devices demonstrated here underscores their broad application prospects in wearable systems and self-powered electronic platforms. Finally, we conclude by discussing the challenges and future prospects in the field of flexible optoelectronic devices based on two-dimensional materials.

## 1. Introduction

The rapidly evolving technological landscape is driving intense demand for innovative, intelligent electronic products. To cater to today’s diverse and dynamic living scenarios, next-generation optoelectronics must deliver not only high performance but also mechanical adaptability. In this context, flexible optoelectronic devices have garnered significant research interest for their outstanding performance and high flexibility, overcoming the rigidity limitations of traditional counterparts [1,2,3,4]. These devices play a crucial role in wearable electronics and self-powered systems, promising more comfortable and convenient user experiences.

Conventional silicon-based optoelectronics face substantial challenges in flexible applications. Their inherent brittleness makes them prone to fracture or performance degradation under bending or stretching, which severely limits their use in flexible formats [5,6]. Furthermore, while semiconductor manufacturing approaches physical limits, silicon devices encounter performance bottlenecks due to quantum effects. These constraints have motivated the exploration of alternative materials.

With their atomically thin structures and exceptional electrical and mechanical properties, two-dimensional (2D) materials represent an ideal choice for flexible optoelectronics [7,8,9,10,11]. The emergence of 2D semiconductors like graphene and TMDs, along with conductive materials like MXenes, has opened up new avenues. Their inherent mechanical flexibility and stable optoelectronic characteristics under deformation ensure reliable operation, injecting new momentum into fields such as wearable technology, flexible displays, and smart nanosystems.

This review provides a systematic overview of the progress in applications of three typical 2D materials in flexible optoelectronics. It delves into their performance in specific devices, analyzing their unique physical properties to explain the mechanisms behind strain-tolerant bandgaps and efficient optoelectronic conversion. This work aims to offer valuable insights for the future development of flexible electronic technologies.

## 2. Fundamental Properties of Key 2D Materials

### 2.1. Graphene

As a two-dimensional material with the thickness of a single atomic layer, graphene combines exceptional mechanical strength with unique optoelectronic properties, making it a cornerstone for flexible electronics. Its high optical transparency (~97.7%), coupled with its exceptional electrical conductivity (resistivity as low as 10^−6^ Ω·cm) and high carrier mobility, underpins its role as an ideal flexible transparent electrode. Crucially for flexible applications, graphene possesses a remarkably high Young’s modulus (~1 TPa) and intrinsic strength (~130 GPa), enabling it to withstand significant mechanical strain without fracture [12]. However, in devices, practical strain tolerance is often limited by defects and grain boundaries, which act as stress concentrators and crack initiation sites, highlighting the importance of synthesizing high-quality films for durable flexible electrodes [13]. These integrated properties establish its physical foundation for application in tunable flexible optical modulators and field-effect transistors, where its conductivity and flexibility are simultaneously leveraged.

### 2.2. Transition Metal Dichalcogenides (TMDs)

Transition metal dichalcogenides (TMDs) exhibit a defining layer-dependent property: as thickness reduces to a monolayer, their band structure transitions from indirect to direct. This dramatically enhances photoluminescence efficiency, making monolayers like MoS_2_ (direct bandgap ~1.8 eV) exceptional candidates for the active layer in flexible photodetectors, LEDs, and ultrathin photovoltaics. Crystal phase engineering further tunes these properties, with the semiconducting 2H phase suited for active layers, and the metallic 1T/1T′ phases proving promising for flexible electrodes. Flexible devices’ mechanical behavior under strain is critical. Monolayer MoS_2_ exhibits a high Young’s modulus (~270 GPa) but a relatively low fracture strain (6–11%) [14]. Multilayer stacks or heterostructures can provide superior flexibility through interlayer sliding, which dissipates strain energy and delays crack propagation [15]. The presence of defects and grain boundaries can significantly reduce mechanical strength, emphasizing the need for controlled growth and passivation in fabricating reliable flexible devices [16].

### 2.3. MXenes

With a general formula M_n+1_X_n_T_x_ and tunable surface groups, MXenes are versatile for flexible optoelectronics. They combine high electrical conductivity with good visible light transmittance, making them promising candidates for replacing indium tin oxide as flexible transparent electrodes. This potential has been concretely demonstrated in recent studies. For instance, highly conductive and transparent Ti_3_C_2_T_x_ MXene inks have been successfully deployed in fabricating mechanically robust, flexible micro-supercapacitors, showcasing their direct applicability in printed energy-storage components for wearable electronics [17]. Furthermore, hybrid electrodes composed of MXene-dressed Ag nanowire networks have been developed; these not only maintain high transparency and conductivity but also significantly enhance the efficiency and operational stability of optoelectronic devices, providing a viable high-performance alternative to ITO [18]. Their tunable function facilitates optimal energy level alignment in device stacks.

The mechanical and interfacial properties of MXene films are crucial for reliable integration. The flexibility and fracture toughness of macroscopic films depend on flake architecture, while strong interfacial adhesion to polymer substrates is essential in preventing delamination during bending. This can be enhanced through surface group engineering or binder use [19]. Strategies like nanofiber compositing have been shown to significantly improve the mechanical durability and bending cycle stability of MXene-based flexible electrodes [20].

## 3. Preparation Methods for 2D Materials

### 3.1. Top-Down Approaches

Preparation methods initiated from the top down have been demonstrated to exfoliate layered bulk materials into single- or few-layer two-dimensional (2D) materials. This process is achieved by disrupting interlayer van der Waals forces in bulk crystals, with the primary methods employed including mechanical exfoliation and ultrasonic liquid-phase exfoliation. Mechanical exfoliation employs mechanical stress to enhance the interlayer spacing of two-dimensional materials, consequently weakening the van der Waals forces [21]. Following Geim et al.’s seminal work on monolayer graphene exfoliation from bulk graphite using adhesive tape, the field of mechanical exfoliation has expanded to encompass a range of other two-dimensional (2D) materials, including transition metal dichalcogenides (TMDs) and MXenes. Although repeated tape exfoliation yields high-quality thin layers with excellent intrinsic properties through a simple process, limitations such as its low yield, small scale, and uncontrollable dimensions hinder its large-scale application in practical devices. In order to address these challenges, researchers have recently developed metal-layer-assisted methods, which rely on strong interactions between metal layers and 2D materials and can prepare large-area, high-quality materials with high controllability and universality. In contrast, ultrasonic liquid-phase exfoliation involves the immersion of bulk materials in a solution. It has been demonstrated that, under ultrasonic agitation, the interlayer van der Waals forces within the dispersed bulk material are weakened, ultimately yielding few- or monolayer 2D materials [22]. This method facilitates large-scale material production in a liquid phase environment. Among these, ion intercalation exfoliation employs electrochemical or chemical methods to insert ions between layers, thereby expanding interlayer spacing. Subsequent ultrasonic treatment yields high-concentration 2D material dispersions. However, this method typically introduces a significant number of defects and contaminants into the material, making it challenging to precisely control the number of layers and material area. In recent years, significant advances have been made in the field of selective exfoliation technology, leading to notable progress in specific transition metal dichalcogenide synthesis. By regulating the type and concentration of intercalated ions, it is possible to selectively prepare a single type of two-dimensional material, which in turn provides new insights for large-scale production.

Top-down preparation methods, which exfoliate layered bulk materials into single- or few-layer two-dimensional (2D) materials by disrupting interlayer van der Waals forces, primarily include mechanical and liquid-phase exfoliation. Mechanical exfoliation leverages stress to weaken these forces. Since Geim et al.’s seminal work on the adhesive-tape exfoliation of monolayer graphene, this approach has been extended to other 2D materials like transition metal dichalcogenides (TMDs) and MXenes. While it produces high-quality layers, its low yield and poor scalability limit its practical application in devices. To address this, researchers have developed metal-layer-assisted methods, leveraging strong interactions for more controllable, large-area preparation. A notable recent advancement is the optimization of Au-assisted TMD exfoliation using (3-aminopropyl)triethoxysilane (APTES), which enables reliable large-area monolayer production. This development constitutes significant progress toward scalable, high-quality material synthesis for optoelectronics [23].

In contrast, liquid-phase exfoliation involves the immersion of bulk materials in a solvent, where ultrasonic agitation weakens interlayer forces to produce dispersed few- or monolayer flakes. This method facilitates larger-scale production. Among the various techniques available, ion-intercalation exfoliation expands interlayer spacing via ion insertion (electrochemical or chemical) followed by ultrasonication, yielding high-concentration dispersions. However, it often introduces defects and offers limited control over layer number and flake size. Significant advances have been made in selective exfoliation via the regulation of intercalant type and concentration, enabling the targeted synthesis of specific 2D materials. Furthermore, high-throughput liquid-phase methods have emerged as pathways that are directly relevant for flexible, scalable optoelectronics. For instance, wet-jet milling has been demonstrated to be an efficient technique of producing large volumes of 2D crystals (e.g., graphene) and printable inks suitable for flexible electronics, as evidenced by their subsequent use in fabricating screen-printed, mechanically compliant micro-supercapacitors [24,25]. These developments in scalable top-down processing—particularly optimized metal-assisted exfoliation and high-yield liquid-phase functional ink production—are crucial for advancing the manufacturing of practical, large-area flexible optoelectronic devices.

### 3.2. Bottom-Up Approaches

The bottom-up approach facilitates the regulated synthesis of two-dimensional materials through precisely manipulating nucleation and growth processes at the atomic scale. The present study categorizes the subject into bottom-up van der Waals epitaxial growth methods, such as solution-based techniques, physical vapor deposition (PVD), and chemical vapor deposition (CVD). Solution-based methods induce nucleation within specific solution environments and control-oriented growth, ultimately precipitating two-dimensional materials. This method offers certain advantages, including ambient-pressure low-temperature operation, simplified process flow, diverse material systems, and relatively low production costs. However, the resulting materials frequently exhibit issues such as high defect density, doping effect susceptibility, and insufficient structural integrity. Physical vapor deposition (PVD) is a process that involves solid precursor sublimation upstream in order to convert them into a gaseous state. These precursors are then transported by carrier gas and undergo recrystallization and deposition on the substrate surface in a downstream low-temperature region [26]. This technology is predicated on physical phase transformation reconstruction as opposed to chemical reactions; as such, it offers significant advantages in terms of controlling product morphology and structure. Conversely, chemical vapor deposition (CVD) involves the induction of gas-phase chemical reactions between precursors at elevated temperatures to yield the desired target products. This process bears a resemblance to physical vapor deposition (PVD), and is followed by carrier gas-driven transport to the low-temperature substrate surface for deposition process completion [27]. In this method, parameters such as precursor type, reaction temperature, system pressure, gas flow rate, and substrate type collectively influence the kinetic and thermodynamic behavior of two-dimensional material growth. By meticulously regulating these parameters, the thickness and area of various two-dimensional materials—including graphene and transition metal dichalcogenides (TMDs)—can be synthesized in a precise manner.

Flexible optoelectronic device fabrication imposes stringent requirements on material synthesis. Bottom-up growth methods must reconcile the need for high-quality two-dimensional materials with the thermal and mechanical constraints inherent to polymer substrates. Critically, the synthesis route dictates key film characteristics including defect density, grain boundaries, and interfacial adhesion, which collectively govern device performance and resilience under cyclic bending. Solution-based methods rely on chemical reactions in liquid phases for material assembly. Their principal advantage lies in their compatibility with low-temperature processing, enabling direct film formation on heat-sensitive substrates such as polyimide. For instance, considerable-quality MoS_2_ monolayers have been achieved on polyimide at temperatures of around 300 °C [28]. A notable drawback, however, is that solution-processed films can suffer from inconsistencies in morphology and elevated defect concentrations, potentially compromising mechanical robustness [29]. Addressing these limitations through precursor design and process optimization is therefore essential. From an application standpoint, the scalability of these techniques is a major benefit. The inkjet printing of functional graphene inks, for example, has proven effective for patterning flexible transparent conductive tracks [30].

Due to its near-ambient temperature operation, physical vapor deposition (PVD), which is exemplified by sputtering, offers excellent substrate choice flexibility. This allows for the direct coating of delicate polymer foils without thermal degradation. The mechanical integrity of the resultant film—meaning its adhesion to the substrate and resistance to cracking—is intensely sensitive to deposition parameters. Strategies such as ion-beam substrate pretreatment are commonly employed to strengthen this interfacial adhesion [31]. PVD is predominantly utilized for depositing conductive metal oxide layers (e.g., ITO) in flexible devices. A persistent challenge remains: mitigating the inherent brittleness of these ceramic materials to ensure reliable operation under repeated mechanical stress. Chemical vapor deposition (CVD) yields the highest crystalline quality for materials like graphene and transition metal dichalcogenides (TMDs) over large areas. Its primary limitation for flexible electronics stems from its excessively high growth temperatures (often >600 °C), mandating a separate transfer step to a flexible host. This transfer process is arguably the most critical determinant of final device performance, as it can induce detrimental defects such as cracks, folds, and polymer residues that degrade both the electronic and mechanical properties [32]. Consequently, significant research efforts have been dedicated to perfecting low-impact transfer methodologies. Despite this hurdle, successful demonstrations have validated the approach, i.e., integrating transferred CVD graphene into robust transparent electrodes and employing CVD-grown MoS_2_ in high-performance flexible transistors [33].

## 4. Applications in Flexible Optoelectronic Devices

### 4.1. Flexible Photodetectors

Flexible photodetectors, featuring outstanding mechanical flexibility and deformation adaptability, are optoelectronic conversion devices built on flexible substrates. The primary function of these devices is to transform incident light signals into recognizable and processable electrical signals [1]. In comparison to conventional photodetectors that utilize rigid semiconductor materials, they offer substantial advantages in specialized applications, including wearable devices, conformal coating systems and implantable medical devices. Such devices are notable for their ultra-thin structure, lightweight nature, and exceptional mechanical properties, including bend-, stretch-, and foldability. It is suggested that these devices have significant application value in fields such as wearable imaging sensing, biomedical monitoring, and portable optical communication [34,35].

Flexible photodetectors’ operational principles primarily rely on three mechanisms: the photoconductive, photovoltaic, and photothermal effects. The first of these is defined as the phenomenon in which conductive or semiconductor materials generate additional charge carriers following the absorption of photons with energy exceeding their bandgap. This results in an increase in electrical conductivity. The process necessitates adequate incident photon energy to initiate intrinsic or impurity absorption. The primary distinction from the photovoltaic effect is the need for an external electric field to effectively separate photo-generated charge carriers, thereby amplifying the output photocurrent signal. As described by Einstein, the photovoltaic effect is the phenomenon whereby a semiconductor absorbs photon energy to generate charge carriers (electron-hole pairs). These carriers separate under the influence of an internal electric field, forming a photocurrent. Such detectors have been shown to exhibit low dark current and high detection efficiency. However, it is important to note that their responsivity is typically lower than those based on the photocurrent effect. When the applied electric field is aligned with the internal one, the charge carrier separation efficiency and device response speed can be enhanced. It has been established that, at elevated reverse bias voltages, photodiodes undergo avalanche multiplication or breakdown. The device achieves carrier multiplication through collision ionization, thereby providing greater photoconductive gain [36], a mechanism that renders it highly valuable for low-light detection applications. The photogate effect is a specialized form of the photocathode effect. The generation of an equivalent gate voltage is achieved through the selective trapping of photo-generated carriers by defect states, which regulates channel conductivity. This mechanism endows detectors with high responsivity and low response speed, rendering them suitable for high-sensitivity detection applications. The photothermal–electric effect is defined as a photoconversion mechanism based on photothermally induced temperature gradients. When materials are exposed to non-uniform illumination, a temperature difference (ΔT) is established within the semiconductor, thereby inducing a thermoelectric voltage (VPTE) via the Seebeck effect. This voltage is expressed as VPTE = (S1 − S2)ΔT, where S1 and S2 are the material’s Seebeck coefficients [37]. This effect drives carrier motion under zero bias voltage conditions; however, the generated voltage is typically small (in the micro- to millivolt range). It is therefore imperative that a high-quality ohmic contact be established at the metal–semiconductor junction, in order to facilitate current conduction through the device.

Beyond the application of pure 2D materials, the construction of zero-/two-dimensional (0D–2D) hybrid architectures (e.g., combining nanocrystals or perovskites with 2D semiconductors) has emerged as a powerful strategy for tailoring photodetector performance. The interfacial physics—particularly the energy and charge transfer processes—plays a decisive role in these systems. For instance, systematic studies have elucidated how semiconducting nanocrystals (like CsPbBr_3_) in close proximity to transition metal dichalcogenide (TMD) monolayers (like MoSe_2_) can engage in efficient non-radiative energy transfer. This process can generate free carriers within the 2D layer itself, offering an alternative and potentially superior pathway for enhancing photocurrent generation compared to direct photoexcitation and charge separation [38]. Integrating such a quantitative understanding of interfacial dynamics is crucial for rationally designing next-generation hybrid flexible photodetectors with tailored spectral responses and enhanced gain. In the field of flexible photodetector research, several representative studies have demonstrated the application potential of two-dimensional (2D) materials. To capture the broader landscape, significant advancements have been made in large-area devices based on MoS_2_ and graphene/TMD heterostructures on flexible substrates; pioneering works in this regard include graphene/MoS_2_ photodetectors with high responsivity over large areas [39] and wafer-scale homogeneous MoS_2_ layers on plastic for high-performance detection [40]. This line of research has since expanded, encompassing a range of innovations including transparent MoS_2_/mica architectures [41], N-doped MoS_2_ on elastomers [42], self-powered Sb_2_Te_2_/MoS_2_ broadband detectors [43], and devices with backside mirrors for photocurrent enhancement [44].

**Figure 1:**
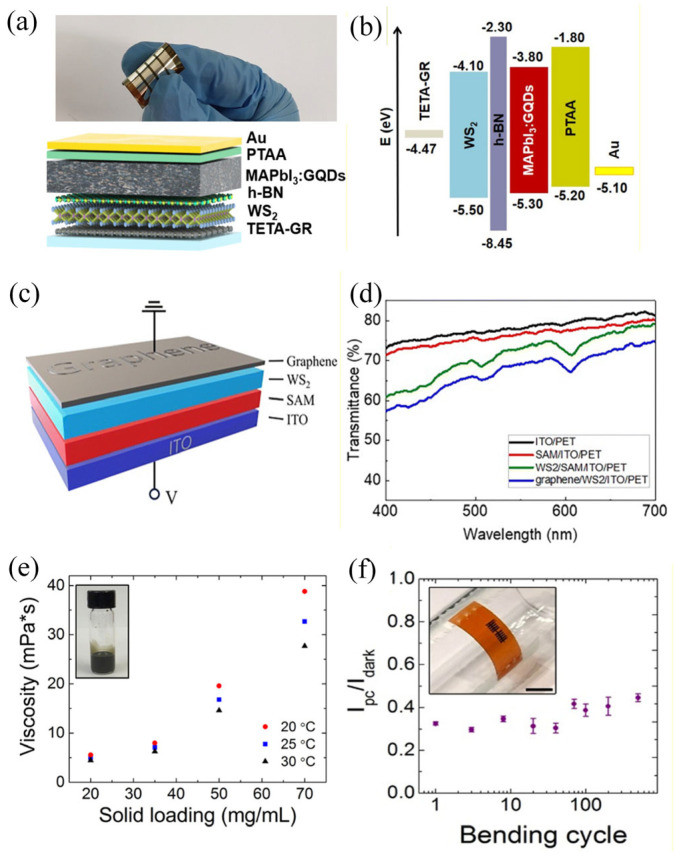
(**a**) Device structure/real image [45]. (**b**) Energy band diagram of a typical TETA-GR/WS_2_/h-BN/MAPbI_3_:GQDs/PTAA/Au PD [45]. Copyright 2025 by the American Chemical Society. (**c**) Schematic illustration of the fabricated photodetector with a self-assembled monolayer (graphene/WS_2_/SAM/ITO) [46]. (**d**) Optical transmission spectra of each layer in the photodetector [46]. Copyright 2025 by the Royal Society of Chemistry. (**e**) Photograph of the MoS_2_/EC ink (inset) and its viscosity dependence on solid loading and temperature [47]. (**f**) Bending test over 500 cycles showing invariant sensitivity. Inset: photograph of the flexible MoS_2_-Gr device. The scale bar is 3 mm [47]. Copyright 2019 by the American Chemical Society.

Jang, Chan Wook et al. successfully fabricated flexible perovskite photodetectors with high detection efficiency and excellent stability by synergistically utilizing multiple 2D materials, including doped graphene, graphene quantum dots, WS_2_, and h-BN, as shown in Figure 1a,b [45]. Subsequently, Meng-Ching Lai et al. developed an ultra-thin, flexible, self-powered transparent photodetector based on all-two-dimensional materials and a polar molecular layer (Figure 1c), achieving a responsivity of 1.58 mA W^−1^ at zero bias and maintaining stable performance after 150 bending cycles (Figure 1d) [46]. Research by Seo, Jung-Woo Ted et al. demonstrates a scalable manufacturing approach for fully inkjet-printed photodetectors. Their method employs molybdenum disulfide nanosheets as the active material and graphene as the electrode, utilizing an ethyl cellulose ink formulation to produce permeable films with high conductivity. In their study, they successfully fabricated two device types: thermally annealed devices on glass substrates achieved rapid optical response times of 150 µs, while photo-annealed devices on flexible polyimide substrates attained high optical responsivities exceeding 50 mA W^−1^ (Figure 1e,f). Curing time was concurrently reduced to the millisecond range, and devices maintained functional stability after enduring over 500 bending cycles [47]. Through distinct technological approaches, these studies demonstrate the application potential of two-dimensional materials in flexible photodetectors.

Furthermore, solution-processed monochalcogenides have emerged as a highly relevant material family for scalable and flexible photodetection. Liquid-phase exfoliated InSe enables highly sensitive photodetectors [48], while GaSe facilitates light-driven thin-film transistors [49]. Versatility is further shown by GaS nanoflake-based UV-selective photoelectrochemical detectors [50], and solution-processed InSe/ITO hybrid films for aqueous media detection [51]. Notable work that further substantiates the promise of specific 2D material families as flexible transparent electrodes includes the use of bio-inspired MXene electrodes. For instance, Chen et al. developed a flexible UV photodetector employing a transparent MXene electrode, which demonstrated high mechanical durability and stable optoelectronic performance under bending, providing a concrete device-level example of MXenes’ potential in next-generation flexible optoelectronics [52]. These studies demonstrate the application potential of two-dimensional materials in flexible photodetectors through distinct technological approaches.

Summarizing six representative examples, Table 1 compares the performance of 2D material-based flexible photodetectors across key parameters: device description, active material, responsivity, and bending performance. The responsivity data reveals extensive variation, from 1.58 mA W^−1^ to approximately 10^11^ mA W^−1^, highlighting the distinct capabilities of different material systems such as all 2D stacks and graphene heterostructures. Mechanical flexibility is indicated through the bending cycles (e.g., 150, >500) or radius (5 mm, 10 mm), although one entry only specifies the use of a flexible PET substrate. Arranged for clarity, this table serves as a direct reference for benchmarking the optoelectronic efficiency and flexible durability of current device designs.

### 4.2. Flexible Light-Emitting Devices

In the field of flexible optoelectronics, light-emitting devices primarily achieve luminescence through two distinct physical mechanisms: electro- and photoluminescence. The former relies on carrier transport and recombination mechanisms, where an electric field applied across the device injects electrons and holes [55]. These carriers ultimately recombine within the light-emitting layer, generating radiative photon emission. In contrast, photoluminescence relies on the interaction between an external excitation light source and the luminescent material, prompting the luminescent centers within it to undergo a radiative transition from an excited to a ground state. These two distinct luminescence mechanisms dictate that flexible light-emitting devices require fundamentally different technical approaches in terms of material selection, structural design, and performance optimization.

The development of flexible electroluminescent devices focuses on addressing the stability issues of traditional rigid structures when subjected to complex mechanical deformations such as bending, stretching, and twisting. This necessitates a systematic restructuring at the physical level, encompassing key technological aspects including the development of novel flexible electrode materials, the optimization of interfacial engineering between functional layers, and improved encapsulation processes. Attention must also be paid to maintaining stable carrier injection balance and efficient recombination efficiency under dynamic deformation conditions. While flexible photoluminescent devices avoid the complexity of carrier injection management, they still face challenges such as quantum efficiency luminescent center decay and changes in excited-state lifetime under sustained mechanical stress [56]. The unique advantage of these devices lies in their simplified structure, eliminating the need for complex multilayer film systems and electrode configurations. They also offer broader material selection and more flexible structural design possibilities. These two technological approaches complement each other well in terms of material systems, device configurations, and performance characteristics. Together, they drive flexible light-emitting technology toward higher performance and greater adaptability, providing crucial technical support for innovative applications such as wearable electronics and flexible displays.

**Figure 2:**
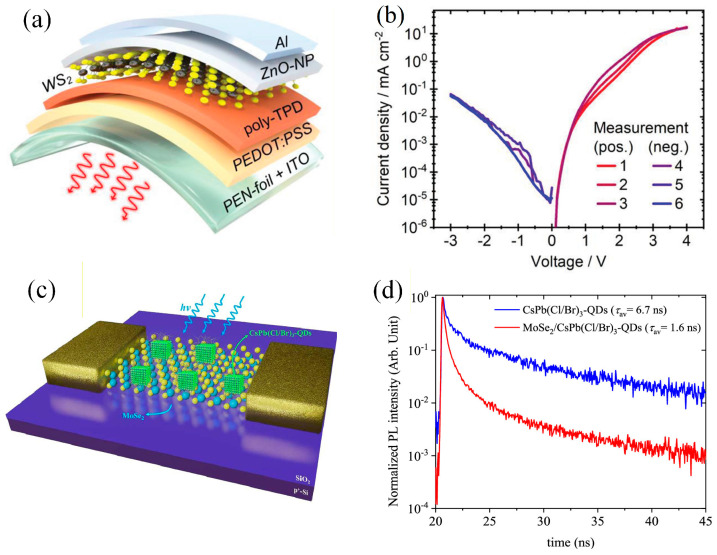
(**a**) p–n device architecture on ITO-coated foil substrate with monolayer WS_2_ synthesized by metal organic chemical vapor deposition (MOCVD) as the active material, organic hole injection (PEDOT:PSS) and hole transport layers (poly-TPD), and a ZnO-NP layer on the cathode side [57]. (**b**) I-V characteristics measured three times in forward and reverse bias, respectively, showing a rectifying ratio above 100 at ±3 V [57]. Copyright 2022 by Advanced Optical Materials. (**c**) Schematic of the MoSe_2_/CsPb(Cl/Br)_3_-QDs hybrid FET under incident light [58]. (**d**) Schematic of the MoSe_2_/CsPb(Cl/Br)_3_-QDs hybrid FET under incident light [58]. Copyright 2020 by the American Chemical Society.

In the domain of flexible optoelectronic devices, numerous representative studies have demonstrated the extensive application prospects of two-dimensional materials. Andrzejewski et al. created the first large-area flexible light-emitting device based on two-dimensional materials, as demonstrated in Figure 2a [57]. This was accomplished by integrating transition metal dichalcogenide monolayers, grown via metal-organic chemical vapor deposition, onto conductive polymer substrates to form p-n junctions. This enabled uniform red light emission over several square millimeters, as well as the first 30 meV electroluminescence energy tuning via bending strain, as demonstrated in Figure 2b [57]. Furthermore, Lee et al. significantly enhanced the optical response characteristics of devices by integrating green-emitting CsPbBr_2_I_1_ and blue-emitting CsPb(Cl/Br)_3_ quantum dots with two-dimensional MSe_2_ field-effect transistors. Their study confirmed that quantum dot hybridization (two-dimensional materials) induced fluorescence quenching. The p-channel current in the hybrid device decreased markedly, while the n-channel photocurrent and light response rate increased substantially. The photoinduced n-type doping phenomenon can be attributed to the photogating effect generated by trap states, which are introduced by perovskite quantum dots (Figure 2c,d) [58]. These studies help advance flexible optoelectronic device technology from a variety of perspectives.

Table 2 provides a structured comparison of five distinct flexible light-emitting devices, detailing their descriptive identifiers, operational emission mechanisms (electroluminescence, EL, or photoluminescence, PL), core active materials, and resultant emission colors. It encompasses a technologically diverse selection, ranging from two-dimensional semiconductors (e.g., WS_2_ monolayers and MSe_2_-based hybrids) and intrinsically stretchable polymers to inorganic III-V micro-LEDs and polymer/2D material composites. The spectrum of emitted light—covering red, green/blue, and specific blue hues—illustrates the color versatility achievable within flexible platforms. This comparative framework facilitates a clear evaluation of the foundational material choices and operating principles across different flexible optoelectronics development pathways.

### 4.3. Flexible Optical Modulators

As a key component for regulating optical signals in flexible optoelectronic systems, the core function of flexible optical modulators lies not in their active generation of photons, but also in dynamically manipulating the key parameters of transmitted light waves—including amplitude intensity, phase delay, polarization state, and propagation path—through external stimuli such as electric and thermal fields or mechanical strain. This enables the switching, modulation, and routing of optical signals, supporting the coordinated operation of downstream modules like flexible optical sensing and communication. The core challenge currently faced by researchers in this field lies in the tendency of flexible devices to induce physical waveguide structure deformation or changes in interfacial stress distribution during mechanical deformations such as bending, folding, or even stretching [61]. This ultimately leads to performance instability issues like modulation rate fluctuations and increased insertion loss. The physical core of addressing this challenge lies in precisely controlling the waveguide material equivalent refractive index through external fields. These indirectly adjust the propagation characteristics of light waves within the waveguide by altering the material’s dielectric constant, carrier concentration, or waveguide geometry, thereby enabling effective control over optical parameters. Based on their underlying physical mechanisms, flexible optical modulators can be categorized into three primary types: Firstly, electro-optic modulators, as exemplified by graphene heterojunction devices, rapidly adjust the carrier properties of two-dimensional materials via electric fields to alter the equivalent refractive index, offering fast response times [62]. Secondly, thermo-optic modulators often employ composite structures of phase-change materials and flexible waveguides. These induce structural transitions in phase-change materials via thermal fields, achieving significant changes in the equivalent refractive index. Thirdly, strain-optic modulators are represented by corrugated flexible waveguides, which rely on mechanical strain to adjust its geometric shape or the material’s photoelastic coefficient [63], indirectly achieving equivalent refractive index control.

**Figure 3:**
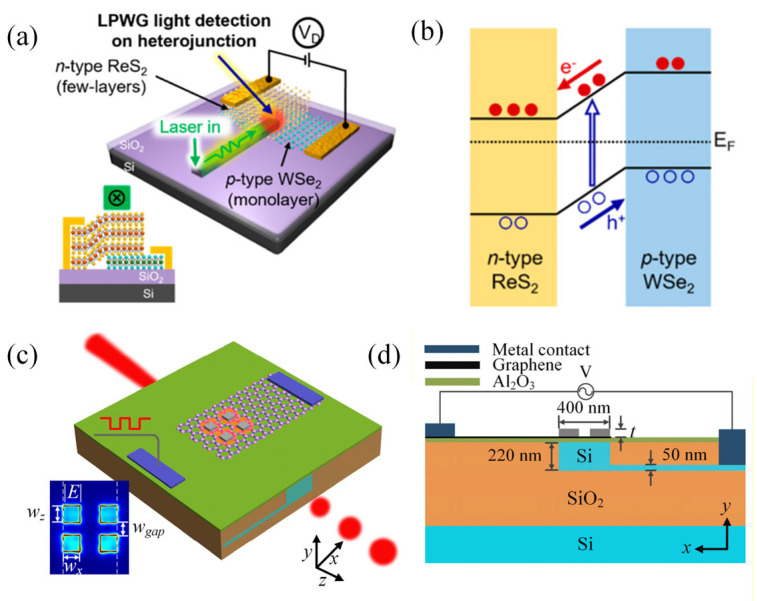
(**a**) Schematic of few-layer ReS_2_/1L-WSe_2_ hetero junction with LPWG (inset shows side view of device), (**b**) working mechanism [64]. Copyright 2023 by the American Chemical Society. (**c**) Three-dimensional and (**d**) cross-sectional views of the proposed waveguide electro-optic modulator integrated with metal patches and graphene. The outer edges of the patches along the z direction are aligned with the edge of the waveguide, where the edge spacing is 400 nm. The gap between patches in the z direction is wgap, and the thickness of the metal patches is t. wx and wz are the widths of the patch in the x and z directions, respectively [65]. Copyright 2018 by Optical Publishing Group.

In recent years, we have witnessed significant advancements in material systems and control mechanisms within this field, with breakthroughs providing a solid foundation for developing next-generation reconfigurable flexible photonic devices. In their study, Kim et al. integrated patterned polymethyl methacrylate waveguides with two-dimensional van der Waals material devices to construct exciton-signal-based optoelectronic circuits. In the presence of an external electric field and heterojunction control, exciton switching, phototransistors and waveguide photovoltaic devices were achieved, as demonstrated in Figure 3a,b [64]. At the same time, Baohu Huang et al. addressed the weak interaction between light fields and single atomic layers in graphene modulators by proposing a novel structure based on localized plasmon enhancement. The application of an ultrathin plasmonic patch in conjunction with voltage-tunable graphene has been demonstrated to result in substantial transmission intensity modulation. The fabricated device demonstrated a modulation rate of 400 GHz on an active area of 0.2 μm^2^, exhibiting an energy consumption of 0.5 fJ bit^−1^, as illustrated in Figure 3c,d [65]. Existing studies provide a series of innovative solutions for developing high-speed optical communication systems and integrated optoelectronic platforms.

This comparison table (Table 3) outlines the performance characteristics of four distinct flexible optical modulators. The listed devices employ a variety of active materials, including carbon nanotubes (CNTs), two-dimensional heterojunctions, graphene, and an Ag/MXene composite, each leveraging a different physical mechanism for light control. Their operational spectra span a wide range—from the terahertz band (0.3–2.0 THz) to visible red light (660 nm) and the near-infrared communication window (~1550 nm)—with one device demonstrating broadband nonlinear capability. Key modulation metrics are provided as follows: the CNT-based modulator achieves a sub-picosecond response and over 50% depth in the terahertz regime, the polymer-integrated device shows microsecond-scale switching at 660 nm, the graphene modulator offers a high bandwidth of 400 GHz at 1550 nm, and the MXene composite provides a ~10.3% modulation depth via nonlinear saturable absorption. The table thus offers a concise, side-by-side summary of the current technological approaches to achieving optical modulation in flexible formats.

### 4.4. Flexible Solar Cells

Flexible solar cells represent a pivotal technology in developing wearable electronics and building-integrated photovoltaics (BIPV). The advancement of this technology is contingent on synergistic progress in material innovation and structural design. The present research principally concentrates on three core areas: active layer material systems, electrode structures, and interface engineering. Regarding the first of these, the combination of polymer donor materials with non-fullerene acceptors has been shown to significantly enhance device mechanical flexibility and energy conversion efficiency [67]. Recent studies have achieved photovoltaic conversion efficiency levels in excess of 18%, while retaining more than 90% of the initial performance at a bending radius of 1 mm [68]. For perovskite active layers, the incorporation of two-dimensional (2D) materials as functional additives has notably emerged as a promising strategy for improving film quality and optoelectronic properties. Electrode materials have evolved from traditional indium tin oxide (ITO) to include metal nanowire/conductive polymer composite and transparent electrodes. It has been demonstrated that graphene and its derivatives exhibit distinct advantages as transparent electrode materials. The atomic-layer thickness and sp^2^ hybridized orbital structure of these materials lead to excellent conductivity and mechanical flexibility. They significantly improve electrode bend resistance while maintaining low sheet resistance and high transmittance. Interface engineering is a process involving the modulation of electrode work functions through optimized combinations of metal oxides and organic semiconductors. The outcome of this process is enhanced charge selectivity extraction, as well as reduced interfacial recombination losses. The innovative strategy of interface engineering has evolved from traditional material stacking and precise charge behavior regulation at interfaces. In particular, zero-/two-dimensional hybrid structures provide a new dimension for such engineering. For example, hybrid materials formed by combining zero-dimensional MoS_2_ quantum dots with two-dimensional graphene have been successfully used as multifunctional interfacial layers in perovskite solar cells. This structure can effectively optimize energy level alignment, promote charge extraction, and suppress ion migration, thereby helping device efficiency exceed 20% [69]. Furthermore, modifying device interfaces with semiconductor nanocrystals such as CuFeS_2_ has also been proven to simultaneously passivate surface defects, improve carrier dynamics, and enhance device stability under light exposure and humid-heat conditions, providing an effective pathway for creating flexible solar cells with both high efficiency and stability [70]. These interface engineering studies based on 0D–2D hybrid systems signify a deepening from qualitative modification to quantitative physical regulation.

The role of 2D materials in perovskite photovoltaics extends beyond interfacial layers to their use as functional additives within the active layer itself. Integrating 2D materials into perovskite precursor ink can profoundly influence crystallization kinetics and defect formation. A representative example is the use of novel 2D BiTeI as an additive in printable perovskite solar cells, which has been shown to positively modulate crystal growth and passivate defects, enhancing device performance [71]. This additive strategy provides a direct route for improving the morphological and electronic quality of perovskite films. Parallel advancements in material science further enrich the flexible optoelectronics toolkit. Beyond their role as additives, the unique properties of 2D materials like BiTeI are being precisely characterized. For instance, gold-assisted exfoliation has enabled the isolation of BiTeI monolayer flakes that exhibit a strong nonlinear optical response, opening up avenues for their use in photonic and light-management applications [72]. Concurrently, research on solution-processable transparent electrodes—such as those based on transparent conducting metal-oxide nanoparticles—addresses critical challenges in forming high-quality charge transport layers for flexible device stacks [73]. These fundamental studies on material properties and processing underpin the development of next-generation, high-performance flexible solar cells. Collectively, these technological advances are propelling flexible solar cells from laboratory research towards practical applications, demonstrating broad potential in the Internet of Things, power supplies, wearable devices, and portable electronics.

In the specific and critical subfield of flexible perovskite solar cells (PSCs), 2D materials play an indispensable role in reconciling high efficiency with mechanical durability. Advanced interface engineering utilizing 2D materials is key to this progress. For instance, perovskite layer surface modification with MoS_2_ nanosheets—especially when combined with tailored molecular ligands like thiols—effectively passivates defects and strengthens the interface, leading to significant enhancements in both efficiency and bending endurance [74,75].

**Figure 4:**
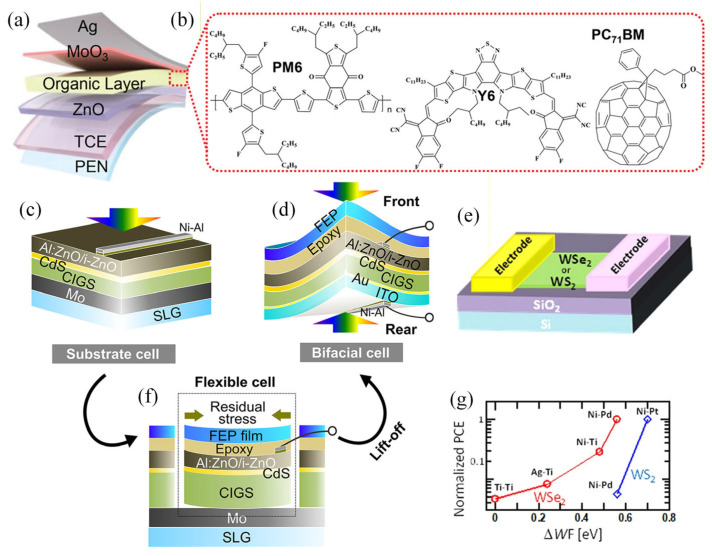
(**a**) Schematic structure of an inverted organic solar cell. (**b**) Chemical structures of organic layers: PM6, Y6, and PC71BM [76]. Copyright 2023 by Advanced Science. Schematic of (**c**) substrate-type, (**f**) flexible-type, and (**d**) light-weight and flexible bifacial-type cell structures via fabrication utilizing the lift-off technique for CIGS solar cells [77]. Copyright 2020 by the American Chemical Society. (**e**) Schematic illustration of the few-layer TMD (WSe_2_ or WS_2_) with an asymmetric electrode solar cell device structure. (**g**) Normalized PCE as a function of ΔWF between the asymmetric electrode pairs for WSe_2_ (red) and WS_2_ (blue) [78]. Copyright 2017 by Scientific Reports.

Kiran A. Nirmal et al. developed flexible transparent electrodes based on an MXene/Ag/MXene structure, achieving 84% transmittance and 9.7 Ω sq^−1^ sheet resistance, which remained stable after 2000 bending cycles. The organic solar cells fabricated with this electrode achieved a conversion efficiency of 13.86% and, for the first time, demonstrated memory and learning functions. They exhibited stable resistive switching characteristics and neuromorphic synapse-like behavior at low operating voltages (±0.6 V), as shown in Figure 4a,b, providing a new pathway and effective solution for smart photovoltaic device development [76]. Nishimura, Takahito’s research proposes an innovative peeling technique utilizing a two-dimensional MoSe_2_ atomic layer as a sacrificial layer, successfully fabricating lightweight, flexible, double-sided CIGS solar cells. The key to this lies in growing a MoSe_2_ layer with its c-axis perpendicular to the Mo surface between the Mo back electrode and the CIGS absorber layer, enabling complete cell structure delamination from the rigid substrate. The delaminated cells achieved an efficiency of 11.5%, maintaining 95% that of the original substrate cells (12.1%). Ultimately, this process demonstrated a highly efficient double-sided power-generating CIGS flexible cell using ultrathin gold and indium tin oxide as the back contact, as shown in Figure 4c,d,f [77]. In proposing a simple, scalable fabrication method, Toshiki Akama’s research team addressed the key challenge of scaling up the application of few-layer transition metal dichalcogenides (TMDs) in manufacturing flexible, translucent solar cells. Using a Schottky-type device structure, the team successfully produced solar cells based on few-layer TMDs with a power conversion efficiency (PCE) of around 0.7%, the highest value yet reported for few-layer TMD solar cells. The device demonstrated significant power generation capabilities on both large SiO_2_ and flexible substrates, validating the method’s substantial potential for mass production. Furthermore, systematic investigations reveal that the PCE and external quantum efficiency (EQE) of the cell strongly depend on different types of photoexcitons (A, B and C), stemming from their distinct carrier dynamics (Figure 4e,g) [78].

Table 4 presents a comparison of the performance of various flexible solar cell architectures incorporating two-dimensional materials. Six representative device types are included, highlighting how 2D materials like graphene, MXenes, and TMDs are deployed in different functional roles, from transparent electrodes and release layers to active and interfacial layers. The corresponding power conversion efficiencies and fabrication methods are listed for each. Notably, the highest efficiencies are achieved in perovskite cells (>20%) and a graphene-electrode cell (>18%), both employing solution processing or transfer techniques. In contrast, devices with TMDs as the primary active layer currently show lower efficiencies (~0.7%), indicating an area for further development. This side-by-side comparison illustrates the material–function performance relationships across different technological pathways.

### 4.5. Flexible Gas Sensors

Based on operating principle, gas sensors can be categorized into semiconductor-type, electrochemical, optical, and field-effect sensors [80]. Electrochemical sensors typically suffer from drawbacks such as long response/recovery times and poor stability. Optical sensors, meanwhile, are constrained by their bulky size, high cost, and lengthy measurement cycles. Although field-effect sensors show great promise, their manufacturing processes are relatively complex, imposing stringent signal amplification requirements [81]. In contrast, metal oxide semiconductor (MOx) sensors stand out for their high sensitivity, rapid response, and compact design. However, the high operating temperatures and rigid structures of MOx sensors limit their application in wearable smart devices requiring flexibility and adaptability.

To overcome these limitations, flexible gas sensors have emerged, effectively filling the technical gap in wearable and portable applications left by traditional gas sensing technologies. These sensors not only operate stably at room temperature but also offer distinct advantages such as lightweight portability, high mechanical flexibility, high system integration, and excellent biocompatibility. These characteristics enable seamless integration with flexible electronic devices, demonstrating significant application value in real-time human health monitoring, rapid food safety screening, and environmental trace gas detection. Further research indicates that, through novel sensing materials and micro/nano-structural designs, flexible gas sensors have surpassed traditional semiconductor-based equivalents in key performance metrics such as sensitivity, gas selectivity, and signal repeatability at room temperature, revealing broad prospects for future development.

**Figure 5:**
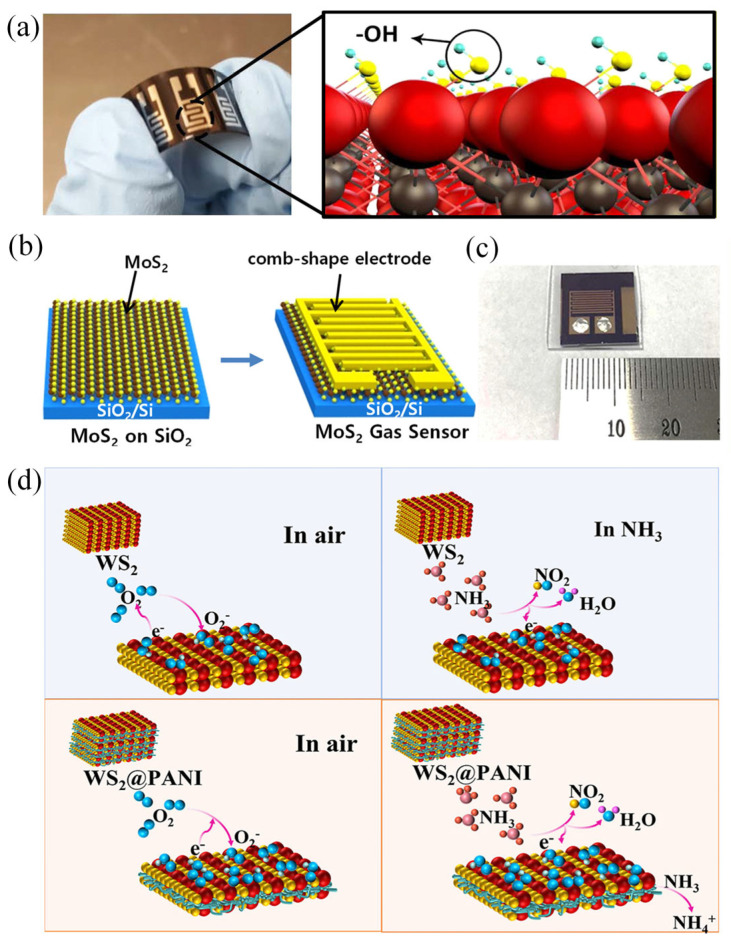
(**a**) Device schematics and physical components [82]. Copyright 2017 by the American Chemical Society. (**b**) Schematic of the fabrication of the MoS_2_ gas sensor. (**c**) Image of the MoS_2_ gas sensor [83]. Copyright 2019 by the American Chemical Society. (**d**) Schematic of the sensing mechanism of the WS_2_@PANI composite-based ammonia sensor [84]. Copyright 2024 by the American Chemical Society.

In recent years, various research teams have achieved significant progress in developing flexible gas sensors based on two-dimensional materials through material innovation and device engineering strategies. Lee, Eunji et al. developed a flexible room-temperature sensor based on Ti_3_C_2_T_x_ MXene nanosheets. Prepared via solution casting, this device effectively detects multiple gases including ethanol, methanol, acetone, and ammonia. Notably, its detection limit for acetone reaches as low as approximately 9.27 ppm (Figure 5a) [82], demonstrating MXene’s advantages in room-temperature multi-gas sensing. Meanwhile, Kim, Youngjun et al. focused on enhancing sensing performance through interfacial regulation. By selecting different electrode materials to modulate the Schottky barrier height formed at the interface with two-dimensional MoS_2_, they demonstrated that increasing the barrier height significantly enhances the sensor’s response to NO_2_ while also improving detection sensitivity for CO and CO_2_, which previously exhibited weak responses (Figure 5b,c) [83]. Furthermore, Wang, Peng et al. addressed the need for ammonia detection in human breath by constructing a heterostructure combining one-dimensional polyaniline (PANI) with two-dimensional WS_2_. This unique design provides abundant gas diffusion pathways and active sites (as shown in Figure 5d), enabling the sensor to achieve a rapid response of up to 216.3% to 100 ppm ammonia with a quick recovery rate [84]. Its excellent interference resistance and stability highlight the practical value of heterojunction engineering in developing high-performance, highly selective wearable sensors. Collectively, these studies—spanning novel material exploration, interface engineering, and heterostructure construction—are propelling two-dimensional material gas sensors toward higher performance, greater selectivity, and enhanced practicality.

Table 5 summarizes the sensing characteristics of four representative two-dimensional (2D) material-based flexible gas sensors. Each entry specifies the device architecture, core sensing material, primary target gas, and its reported response or sensitivity. The 2D materials employed span several key families: MXenes (Ti_3_C_2_T_x_ and V_2_C), transition metal dichalcogenides (few-layer MoS_2_ and WS_2_ nanosheets), and a heterostructure combining WSe_2_ with MXene. These devices are designed to detect different gases, including acetone (C_3_H_6_O), nitrogen dioxide (NO_2_), and ammonia (NH_3_). The performance metrics vary, with one WS_2_-based sensor showing a response of ~216.3% to 100 ppm NH_3_, while a Schottky-barrier engineered MoS_2_ device achieves an approximately threefold enhancement in response to NO_2_ after tuning. This compilation provides a concise, side-by-side comparison of how different 2D material systems are being adapted for flexible gas sensing applications.

### 4.6. Substrate-Limited Compromise

Substrate selection introduces a defining compromise regarding the characteristics attainable with an optoelectronic device. Conventional rigid platforms, like silicon with a silicon dioxide (SiO_2_) layer, facilitate peak intrinsic optoelectronic metrics. Such platforms support high-temperature processes and ultra-smooth surfaces, leading to manifestations like the exceptionally high responsivity (~10^11^ mA W^−1^) in graphene/MoS_2_ photodetector heterostructures [39] and record power conversion efficiencies—exceeding 20%—in perovskite solar cells [69]. However, the inherent cost of this performance is a complete lack of mechanical deformability.

In contrast, the adoption of flexible substrates (e.g., polyethylene terephthalate (PET), polyimide) guarantees this crucial mechanical conformability property. This enables the repeated bending essential for wearable applications, as demonstrated by devices enduring over 500 bending cycles with minimal performance degradation [47]. Acquiring this mechanical functionality typically requires accepting a certain amount of optoelectronic sacrifice. Illustrative examples include the comparatively lower external quantum efficiency observed in intrinsically stretchable light-emitting devices [57] and the reduced modulation depth of optical modulators integrated on flexible polymer waveguides [64] relative to their rigid counterparts. These concessions are not incidental, but directly traceable to flexible materials’ inherent physical limitations: a restricted thermal processing window that compromises material crystallinity, increased surface topography that hinders perfect interfacial contact, and dielectric properties that vary under mechanical strain, potentially affecting operational consistency.

Consequently, advancing flexible optoelectronics demands a paradigm shift. The objective is not simply the transposition of rigid-device designs onto bendable supports. Instead, it requires the holistic and simultaneous optimization of three interconnected domains: high electro-optical efficiency, reliable mechanical robustness, and stable operational performance under dynamic conditions. This framework establishes that decisions regarding mechanical architecture and substrate material are not peripheral considerations but are, in fact, principal factors that demarcate the final performance limits and determine the practical feasibility of flexible optoelectronic technologies.

These trade-offs, and the varied behaviors of devices under strain, are fundamentally rooted in the specific physical response mechanisms of 2D materials integrated on flexible substrates. Their electromechanical behavior is governed by a combination of interrelated mechanisms. Strain-induced lattice deformation modulates carrier mobility through the piezoresistive effect, altering electrical resistance, while non-centrosymmetric crystals like monolayer MoS_2_ generate intrinsic polarization fields via the piezoelectric effect, directly converting mechanical stress into electrical signals. Concurrently, mechanical deformation modifies band structures, influencing charge generation and separation dynamics at heterojunctions. These processes are critically mediated by interfacial properties, where adhesion strength and charge trap density determine response stability under cyclic loading. Ultimately, substrate morphology—including surface roughness and porosity—controls strain distribution uniformity, serving as a decisive factor determining device reproducibility and long-term reliability.

### 4.7. Mechanical Reliability of Flexible Optoelectronic Devices

The performance and reliability of flexible optoelectronic devices are fundamentally governed by the interplay between their electrical, optical, and mechanical properties. Drawing upon the examples cited throughout this review, this section synthesizes a systematic analysis of how mechanical strain influences material physics and device design across different material families.

Firstly, mechanical strain serves as a direct band structure engineering tool in two-dimensional (2D) semiconductors. A clear example is the large-area WS_2_ monolayer light-emitting device, where applied bending strain enabled the active tuning of the electroluminescence peak by approximately 30 meV [57]. This demonstrates that strain can be not merely a source of degradation, but also a functional parameter.

Secondly, the predominant mechanical failure mode in flexible 2D material devices is interfacial delamination, not the fracture of the crystalline sheets themselves. This is a critical limitation for van der Waals heterostructures. Successful device strategies explicitly address this by designing architectures that dissipate stress. For instance, in the WS_2_/PANI heterojunction gas sensor, the one-dimensional polymer network provides a compliant matrix that protects the WS_2_ nanosheets, allowing the device to retain 95% of its response after 1000 bending cycles [84]. Similarly, the fully inkjet-printed MoS_2_ nanosheet photodetector utilizes a porous, permeable film morphology that accommodates strain, contributing to its functional stability after more than 500 bending cycles [47].

Thirdly, long-term reliability under cyclic bending emerges as a decisive metric, revealing a central design trade-off. The excellent durability data from the aforementioned compliant designs (e.g., [47,84]) stand in contrast to the typically unreported or insufficiently characterized mechanical endurance of devices that prioritize ultimate speed or efficiency through complex, rigid nanostructures such as the high-speed plasmonic-enhanced graphene modulator [65]. This contrast underscores how achieving peak optoelectronic performance often comes at the cost of mechanical robustness.

In conclusion, the collective evidence from solar cells, photodetectors, LEDs, and sensors indicates that mechanical reliability must be co-designed at the material and architectural levels. This involves integrating active materials into stress-engineered systems, such as polymer composites or porous networks, to ensure that flexible devices can meet the durability demands of real-world applications.

## 5. Summary and Perspectives

This review has traced the remarkable journey of two-dimensional materials—graphene, transition metal dichalcogenides (TMDs), and MXenes—from fundamental curiosities to foundational components in flexible optoelectronics. Their unique property portfolios have enabled devices that were unimaginable a decade ago. However, as the field matures from demonstrating spectacular individual properties to building reliable systems, a more nuanced and strategic perspective is required. The following discussion aims to provide this, moving beyond a generic recitation of challenges and directly addressing one reviewer’s call for a clearer competitive analysis, actionable insights, and a forward-looking roadmap.

To understand their future, 2D materials must be positioned not in isolation, but within the vibrant and competitive flexible technology ecosystem. When placed alongside organic semiconductors, hybrid perovskites, and amorphous metal oxides, their relative strengths and weaknesses come into sharp focus. The compelling case for 2D materials rests on a triad of definitive advantages: their atomic-layer precision and superior environmental stability, which outperform many solution-processed alternatives; their exceptional intrinsic mechanical robustness, critical for ultra-thin, fatigue-resistant devices, as evidenced by MXene-based electrodes sustaining over 1000 bending cycles; and their unique capacity for van der Waals heterostructuring, enabling “mix-and-match” integration without lattice constraints. However, an honest appraisal must acknowledge the areas in which they currently lag behind. The most significant hurdles remain in scalable, cost-competitive manufacturing and in achieving the routine large-area electronic uniformity of sputtered oxides or printed organics. They therefore show most promise not as a wholesale replacement, but as an enabling technology for next-generation applications. For example, in ultra-conformal bio-sensors, sophisticated wearable systems, or stretchable displays, their extreme thinness, stability, and integration versatility are absolutely essential, justifying their complex fabrication.

From the extensive research compiled, two quantitative, take-home messages emerge with particular force, offering clear guidance for future design. Firstly, the data reveals a fundamental and often inverse relationship between peak optoelectronic performance and mechanical endurance. Devices engineered for record-breaking metrics, like ultra-high responsivity in graphene/TMD heterojunctions, frequently lack robust data on long-term bending stability. Conversely, architectures designed for flexibility from the ground up, such as printed photodetectors, reliably withstand hundreds of bending cycles but with more modest performance figures. This is not a failure but a design principle: the primary goal of the device must dictate the initial material and integration strategy choices from the very beginning. Secondly, and perhaps more critically, a flexible device’s lifetime is almost invariably dictated by its most mechanically vulnerable component. Catastrophic failure usually stems from a brittle electrode cracking or a stiff active layer delaminating the classic “weakest link” phenomenon. At these critical interfaces, the exceptional durability shown by devices incorporating intrinsically tough materials like MXenes offers a powerful lesson: proactively engineering toughness where stress concentrates is a far more effective strategy than retroactively trying to make the entire structure compliant.

Flexible 2D material devices’ transition from laboratory validation to commercial viability critically hinges on aligning material properties with scalable manufacturing methods. A central requirement for economical mass production is the establishment of roll-to-roll (R2R) fabrication platforms, enabling continuous, high-throughput processing. To be compatible with such systems, essential supporting technologies must mature towards clean, residue-free transfer methods that maintain interface quality and maximize production yield, alongside low-temperature processing schemes that prevent damage to affordable polymer substrates like PET. Concurrently, solution-processable printing techniques using 2D material inks present a complementary, directly written strategy for patterning functional components, facilitating the integration of complex circuitry on flexible surfaces. Looking ahead, such a trajectory—from promising prototypes to impactful technology—demands a concerted effort across three escalating tiers of innovation. The foundational tier is advanced material and interface engineering. This involves not only discovering new 2D compounds with tailored properties but also pioneering “gentler” fabrication methods, such as laser-assisted patterning or electrochemical delamination that preserve material quality and ensure robust, defect-free interfaces to prevent delamination. The next tier is device innovation, which is inherent in 2D materials. We must move beyond merely shrinking three-dimensional device concepts. The real opportunity lies in creating novel architectures that leverage quintessential 2D attributes, such as gate-tunable light-matter interaction for ultra-fast optical modulators or exploiting interlayer excitons in heterostacks for novel light-emitting diodes. The ultimate frontier, and the grandest challenge, is true heterogeneous system integration. The future of flexible electronics is not a single perfect material, but a symphony of different technologies working in concert. The key task is to develop the “connective tissue”—reliable flexible interconnects, universal transfer methods, and stable encapsulation schemes—that will allow 2D-material-based sensors, processors, and displays to be seamlessly integrated with silicon microchips, perovskite solar cells, and polymer substrates into multifunctional, robust, and high-performance systems.

In conclusion, the narrative surrounding 2D materials for flexible optoelectronics is maturing from a story of pure promise to one of purposeful implementation. The road forward involves an astute assessment of their competitive niche, adherence to quantitative design rules gleaned from existing research, and the pursuit of a structured roadmap, from fundamental materials to integrated systems. This disciplined approach channels efforts toward applications where these materials offer truly unique solutions. Emerging frontiers—such as flexible neuromorphic devices for on-sensor computing, printed heterojunction architectures for customizable multifunctionality, ultrathin AIoT platforms for imperceptible monitoring, and multimodal deformable sensors for rich environmental decoding—exemplify this targeted evolution. By focusing on these strategic areas, 2D materials can solidify their indispensable role in shaping the future of wearable, conformal, and intelligently responsive electronics.

## Figures and Tables

**Table 1:** A comparison and analysis of two-dimensional material flexible photodetector performance.

Device Description	Core Active Material	Responsivity (mA W^−1^)	Bending
Ultrathin self-powered transparent photodetector [46]	All 2D material stack	1.58	150 bending cycles
Inkjet-printed flexible photodetector [47]	MoS_2_ nanosheets	>50	>500 bending cycles
Large-area graphene/MoS_2_ heterostructure photodetector [39]	MoS_2_	~10^11^	On flexible PET substrate
Liquid-exfoliated InSe based highly sensitive photodetector [48]	Liquid-exfoliated InSe nanoflakes	~10^7^	_
2D Vertical Photodetector competitive with Si [53]	Ti/WSe_2_/Ag vertical stack	200	5 mm
Flexible Self-powered UV Photodetector [54]	ZnO/GQDs heterojunction	254.8	10 mm

**Table 2:** A comparison of two-dimensional material-based flexible light-emitting performance.

Device Description	Emission Mechanism	Active Material	Emission Color
Large-area WS_2_ Monolayer LED [57]	EL	WS_2_ monolayer	Red
QD-2D MSe_2_ Hybrid Device [58]	PL	MSe_2_	Green/Blue
Intrinsically Stretchable PLED [56]	EL	Polymer:PEI	_
Flexible Blue Micro-LED Array [59]	EL	InGaN/GaN	Blue
Stretchable PLED with 2D Additive [60]	EL	Polymer/2D Material Composite	Deep Blue

**Table 3:** A comparison of two-dimensional material-based flexible optical modulator performance.

Device Description	Modulation Rate/Bandwidth	Operating Wavelength/Band	Modulation Depth/Efficiency
Stretchable CNT Terahertz Modulator [64]	~2 × 10^−13^ s	0.3–2.0 THz	>50% (at 0.3–2.0 THz)
Polymer-Waveguide-Integrated 2D Heterojunction Device [65]	Rise: ~8 × 10^−6^ s, Fall: ~1.5 × 10^−5^ s	660 nm	5.8 dB (at 660 nm)
Plasmonic-Enhanced Graphene Modulator [63]	3-dB Bandwidth: 4 × 10^11^ Hz	~1550 nm	_
Ag/MXene Composite Modulator [66]	Nonlinear Saturable Absorption	Broadband	~10.3%

**Table 4:** A comparison of two-dimensional material-based flexible solar cell performance.

Device Type	Core 2D Material	Power Conversion Efficiency (PCE)	Fabrication Method
Perovskite solar cell [76]	MXene Transparent Electrode	13.86%	Solution processing
CIGS Thin-Film Solar Cell [77]	TMDs Release Layer	11.5%	Vapor deposition
TMDs Schottky Solar Cell [78]	TMDs Active Layer	~0.7%	CVD growth
High-Efficiency Flexible Cell [68]	Graphene Transparent Electrode	>18%	CVD transfer
Perovskite Solar Cell [69]	TMDs Interface Engineering	>20%	Solution processing
Flexible Organic Solar Cell [79]	MoS_2_ hole transport layer, Graphene electrode	4.23%	Spin-coating

**Table 5:** A comparison of two-dimensional material-based flexible gas sensor performance.

Device Description	Core 2D Material	Target Gas	Response/Sensitivity
Highly Responsive Flexible Heterojunction Sensor [82]	Ti_3_C_2_T_x_ MXene	Acetone	Not specified
Schottky-barrier engineered FET sensor [83]	Few-layer MoS_2_	NO_2_	Response to NO_2_ enhanced by ~3× (after barrier tuning)
Room-Temperature Multi-Gas Sensor [84]	WS_2_ nanosheets	NH_3_	~216.3% (to 100 ppm)
MXene/TMDs Heterojunction Sensor [85]	WSe_2_/V_2_C MXene heterostructure	NH_3_	Not specified

## Data Availability

All data analyzed or presented in this review are sourced from the publications cited in the reference list.
